# Rabson-Mendenhall Syndrome Nearly Misdiagnosed as Type 1 Diabetes Mellitus: A Case Report

**DOI:** 10.7759/cureus.81190

**Published:** 2025-03-25

**Authors:** Fahad Almotawa, Aziza M Mushiba, Nora Alqahtani, Abdulrahman Mashi

**Affiliations:** 1 College of Medicine, University of Bisha, Bisha, SAU; 2 General Pediatrics, King Fahad Medical City, Riyadh, SAU; 3 Family Medicine, Prince Mohammed Bin Abdulaziz Hospital, Riyadh, SAU; 4 Obesity, Endocrine and Metabolism Center, King Fahad Medical City, Riyadh, SAU

**Keywords:** diabetes mellitus, insulin receptor, insulin resistance, rabson-mendenhall syndrome, rare genetic diseases

## Abstract

Rabson-Mendenhall syndrome (RMS) is a rare genetic condition marked by severe insulin resistance, leading to persistent hyperglycemia that can sometimes be misdiagnosed as type 1 diabetes mellitus (T1DM). This case report details a 34-year-old male who was referred to a tertiary center for genetic evaluation to rule out insulin resistance syndrome. The patient had been diagnosed with T1DM since childhood, struggling to control his hyperglycemia despite high doses of insulin. Physical examination revealed acanthosis nigricans, prognathism, and other dysmorphic features. Genetic testing identified pathogenic variants in the insulin receptor (INSR) gene, confirming the diagnosis of RMS. Insulin resistance syndromes are prone to misdiagnosis, so a thorough patient history and careful physical examination are essential in distinguishing T1DM from insulin resistance syndrome. This is the first documented case of RMS in Saudi Arabia, and we emphasize the clinical findings and genetic confirmation in this patient.

## Introduction

Rabson-Mendenhall syndrome (RMS) is a rare genetic disorder characterized by defective insulin receptors (INSR) and severe insulin resistance. Due to overlapping clinical features, it is often misdiagnosed as type 1 diabetes mellitus (T1DM), with both conditions presenting hyperglycemia and metabolic disturbances [1]. Rabson and Mendenhall first described the syndrome in 1956 while studying three siblings with manifestations of insulin resistance, including acanthosis nigricans and other developmental abnormalities. RMS is an autosomal recessive inherited disease, and its prevalence remains undetermined due to its rarity [2].

Specific data on the incidence of RMS in Gulf Cooperation Council countries - comprising Saudi Arabia, Kuwait, the United Arab Emirates, Qatar, Bahrain, and Oman - are limited due to the condition’s rarity and the lack of comprehensive regional epidemiological studies. However, insights can be drawn from available case reports and genetic studies in the region. For instance, a study published in 2021 documented the first reported cases of RMS in Kuwait, involving a brother-sister pair diagnosed with the condition. This suggests that RMS does occur in the Gulf region, though it remains exceptionally rare [3]. To the best of our knowledge, no RMS cases have been reported from Saudi Arabia. Here, we present the case of a patient who was initially diagnosed with T1DM but was later found to have RMS following genetic investigation.

## Case presentation

A 34-year-old Saudi male with consanguineous parents and a history of T1DM was referred from a secondary hospital for genetic testing and further evaluation of severe insulin resistance and possible lipodystrophy. He had been diagnosed with diabetes at the age of 9 during an incidental checkup, given his strong family history of the condition. Both of his siblings, a brother and a sister, had been diagnosed with diabetes in childhood. Both siblings experienced severe complications - his brother lost his sight, and his sister developed end-stage renal disease. Both siblings passed away in their mid-30s. It was believed they may have shared the same underlying diagnosis. The patient had uncontrolled blood sugar, complicated by diabetic retinopathy, as well as bipolar disorder, for which he received regular psychiatric follow-up.

**Figure 1:**
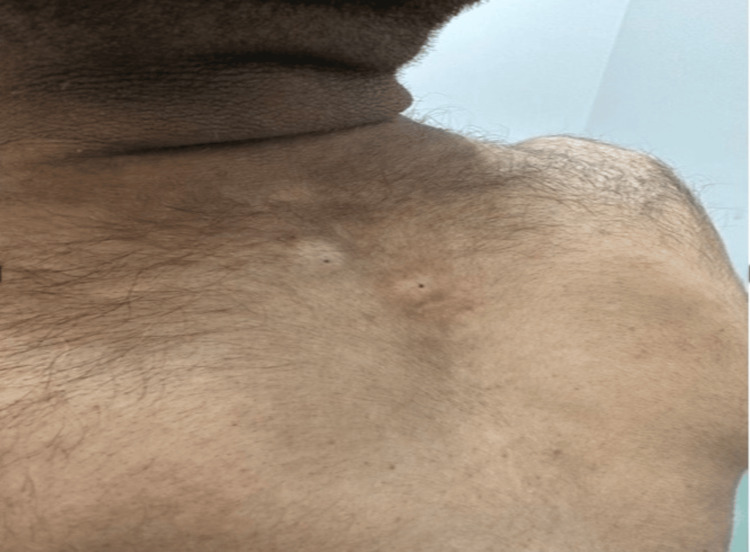
Skin pigmentation of the neck (acanthosis nigricans)

Physical examination revealed that the patient was poorly developed and malnourished. His vital signs were normal, his body mass index was 15.56 kg/m², and his height was 150 cm. Systemic examination did not reveal pallor, icterus, cyanosis, clubbing, lymphadenopathy, or edema. His neck examination was notable for acanthosis nigricans (Figure 1). Prognathism was also observed, though the patient preferred to keep his face concealed.

**Figure 2:**
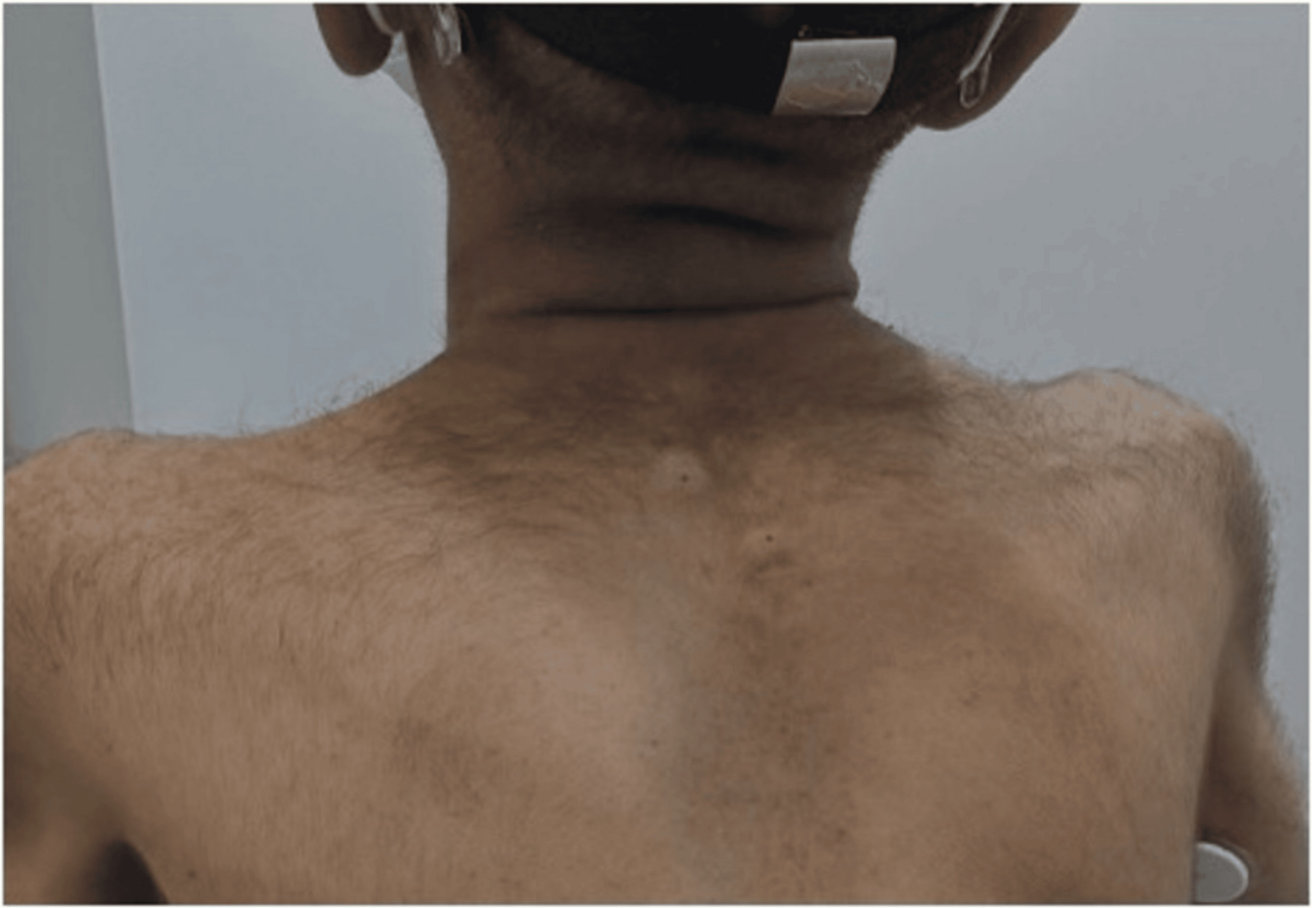
Patient’s back showing cutaneous fat loss

**Figure 3:**
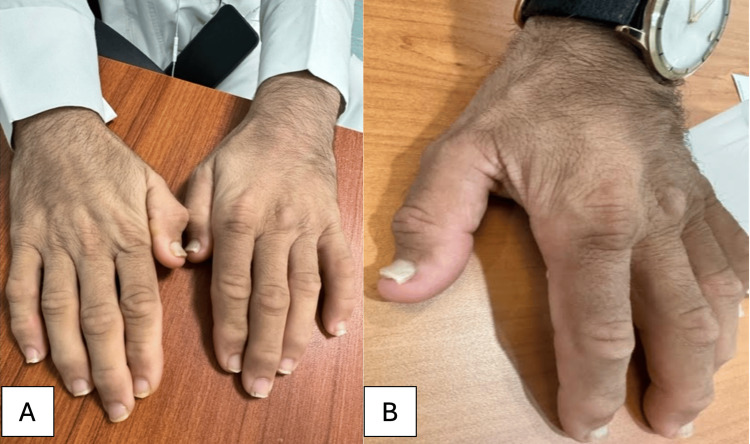
Images showing (A) big, broad thumbs and (B) thick nails

Other notable findings included the loss of cutaneous fat on his back (Figure 2) and large, broad thumbs (Figure 3).

Diagnostic assessment

Genomic DNA was extracted from peripheral blood leucocytes, and whole exome sequencing was performed using CentoXome^®^ (Rostock, Germany). Genetic analysis identified an in-trans biallelic variant in the INSR gene: a hemizygous disease-causing variant (NM_000208.2: c.433C>T p. (Arg145Cys)) and a disease-causing loss of approximately 237 kbp in the 19p13.2 chromosomal cytoband, encompassing exons 1-22 of the INSR gene. These variants were found to be in a compound heterozygous state, leading to a genetic diagnosis of autosomal recessive INSR-related severe syndromic insulin resistance.

**Figure 4:**
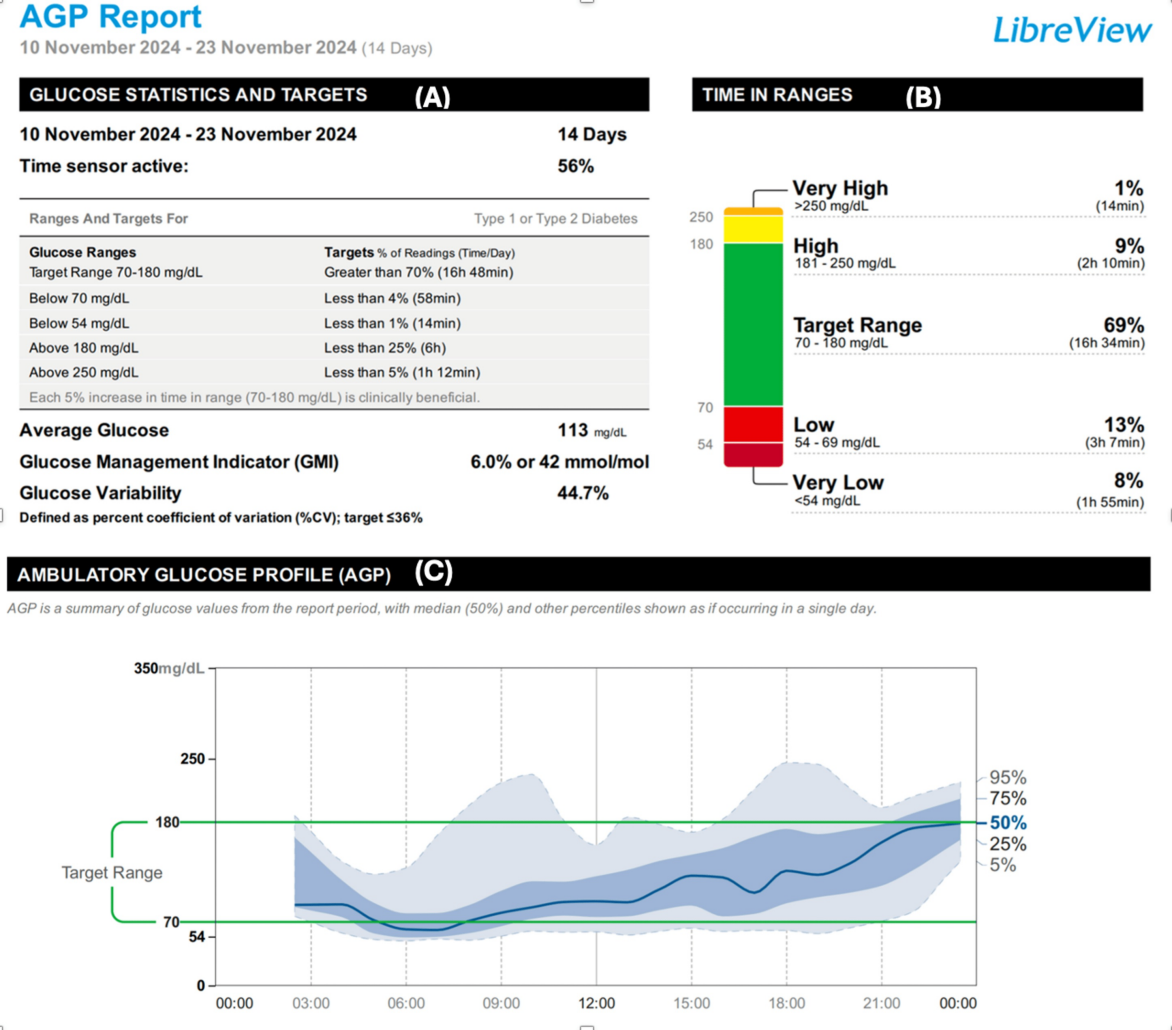
(A) The average glucose and GMI suggest reasonable control of blood glucose, but the high %CV (44.7%) indicates unstable glucose levels. (B) Time in range showing a high TBR, especially in the very low range, reflecting severe episodes of hypoglycemia. (C) The ambulatory glucose profile reveals severe nocturnal hypoglycemia and moderate postprandial hyperglycemia GMI, glucose management indicator; TBR, time below range

The patient achieved excellent glycemic control with high insulin doses (total daily dose of 7.6 units/kg) and metformin. However, strict control of his hyperglycemia resulted in severe hypoglycemia and significant glucose fluctuations, as demonstrated in his ambulatory glucose profile (Figure 4).

Blood tests were performed during the patient’s first visit, with the results shown in Table 1. All tests returned normal results, indicating that renal function, liver function, and complete blood count were within normal limits. His c-peptide level was 971 pmol/L (normal range: 260-1730 pmol/L), and his urinalysis was also normal, showing a glucose level of 50 mg/dL with no ketones present.

**Table 1:** Metabolic profile and routine blood test results of the patient ALP, alkaline phosphatase; ALT, alanine aminotransferase; FT4, free thyroxine; HbA1c, hemoglobin A1c; HDL, high-density lipoprotein; LDL, low-density lipoprotein; TSH, thyroid-stimulating hormone

Test	Result	Reference
HbA1c	6.54	4-5.6%
Fasting glucose	9.3 mmol/L	3.89-5.83 mmol/L
Glutamic acid decarboxylase antibodies	Negative	Negative
C-peptide	971.00 pmol/l	260.00-1,730.00 pmol/l
TSH	1.4 mIU/L	0.3500-4.9400 mIU/L
FT4	12.8 pmol/l	9.00-19.00 pmol/l
Triglycerides	0.68 mmol/L	≤1.70 mmol/L
HDL cholesterol	1.6 mmol/L	>1.55 mmol/L
LDL cholesterol direct	3.39 mmol/L	<2.60 mmol/L
Total cholesterol	5.000 mmol/L	≤5.180 mmol/L
Non-HDL cholesterol	3.40 mmol/L	<3.3 mmol/L
ALT	23.00 U/L	0.00-55.00 U/L
ALP	80.00 U/L	40.00-150.00 U/L
Total bilirubin	9.00 umol/l	3.40-20.50 umol/l
Albumin	41.40 g/l	35.00-52.00 g/l
WBC	4.35 10³/uL	>3.90 to <11.00 10³/uL
Hemoglobin	11.5 g/dL	13.5-18.0 g/dL
Creatinine	74 umol/l	64.00-104.00 umol/l
Calcium corrected, plasma	2.29 mmo/l	2.10-2.55 mmo/l
Microalbumin/creatinine ratio	49.18 mg/g	<30 mg/g

Treatment

The patient was treated with U-300 insulin glargine (80 units) once daily, insulin aspart (70 units with meals), and metformin XR at a maximally tolerated dose of 750 mg daily. Despite being on a large dose of insulin therapy, his blood sugar levels remained uncontrolled, with recurring episodes of profound hypoglycemia and hypoglycemic unawareness, which required hospitalization. His wide glucose fluctuations could be partly attributed to psychosocial factors, including the emotional impact of his deceased siblings, who had struggled with diabetes-related complications. The patient was educated about the potential risks of tightly managing his blood sugar levels and was offered the option of being referred to a mental health specialist.

Outcome and follow-up

After patient education and the use of continuous glucose monitoring, the patient returned to the clinic with improved glycemic control and fewer hypoglycemic events. Pioglitazone was prescribed, but the patient discontinued it due to the development of intolerable bilateral lower limb edema. Empagliflozin was also added to his treatment, but he had difficulty continuing it because of nocturnal polyuria and recurrent urinary tract infections.

## Discussion

RMS is an inherited insulin resistance syndrome caused by mutations in the INSR gene. It is extremely rare and often misdiagnosed as other conditions, such as diabetes mellitus [1]. First described in 1956 by Rabson and Mendenhall, the syndrome was identified in three siblings who exhibited blood glucose fluctuations and insulin resistance [2,4]. The clinical manifestations of RMS are varied, with the most common features being hyperinsulinemia, underweight, acanthosis nigricans, growth retardation, dental anomalies, and hyperthecosis [5]. To our knowledge, this is the first confirmed case of RMS in Saudi Arabia.

RMS results from INSR mutations that reduce insulin receptor function and its affinity for the hormone, leading to insulin resistance in target tissues [6,7]. The specific INSR variant c.433C>T p.(Arg145Cys) identified in this case causes an amino acid substitution from Arg to Cys at position 145 in exon 2 (of 22). According to HGMD Professional 2023.3, this variant has previously been linked to insulin resistance in patients and is considered a founder mutation in the Saudi population [8]. It was classified as pathogenic based on the American College of Medical Genetics and Genomics/Association of Molecular Pathology/Clinical Genome Resource guidelines. The other variant identified was a copy number variant (CNV), a microdeletion that encompasses the INSR gene.

To the best of our knowledge, this is the first time the 19p13.2(7056333_7293912)x1 deletion has been linked to insulin resistance syndromes. The detected loss affects a large 237-kbp region of chromosome 19, including three genes: INSR, MBD3L3, and ZNF557. The CNV was confirmed using qPCR. This deletion removes exons 1-22 of the INSR gene, as well as part of the MBD3L3 and ZNF557 genes. The variants were confirmed to be compound heterozygous, leading to a genetic diagnosis of autosomal recessive INSR-related severe syndromic insulin resistance.

A normal C-peptide level, combined with the absence of anti-glutamic acid decarboxylase antibodies, suggests an alternative diagnosis to T1DM and raises suspicion of insulin resistance syndromes. Physicians, particularly in rural areas, should promptly refer suspected patients for genetic testing to ensure an accurate diagnosis and early intervention.

Insulin resistance is characterized by impaired insulin uptake or response by insulin-sensitive cells, resulting in elevated insulin production without corresponding decreases in blood glucose levels [8,9]. In addition to conventional diabetes medications and dietary management, newer agents such as metreleptin and recombinant IGF-1 have been tested for treating RMS in several case reports. However, they are not yet approved for this indication and are used off-label [10,11].

Treatment of RMS is challenging due to its rarity and the lack of randomized clinical trials to quantitatively assess the efficacy of current therapies [4]. Alternative options to control hyperglycemia include insulin pumps, which may help reduce hypoglycemic events. However, insulin pumps have a limited reservoir capacity of only 300 units, necessitating more frequent replacements of the infusion set, often daily instead of the typical three-day replacement interval.

## Conclusions

To the best of our knowledge, this study presents the first documented case of RMS in Saudi Arabia and establishes the initial link between the microdeletion 19p13.2(7056333_7293912)x1 and insulin resistance syndromes. RMS is extremely rare and can often be misdiagnosed; thus, educating medical practitioners is vital. Early genetic referral and screening can significantly improve patient outcomes. Given the rarity of RMS, current medical interventions primarily focus on symptom management rather than a cure. Family counseling regarding the genetic nature of the disease is a crucial aspect of the treatment plan and can help families prepare for future pregnancies.
